# The Role of Immunotherapy in Extensive Stage Small-Cell Lung Cancer: A Review of the Literature

**DOI:** 10.1155/2019/6860432

**Published:** 2019-11-03

**Authors:** Ioanna Tsiouprou, Athanasios Zaharias, Dionisios Spyratos

**Affiliations:** Pulmonary Department, Aristotle University of Thessalloniki, G. Papanikolaou Hospital, Exohi, Thessaloniki 57010, Greece

## Abstract

Lung cancer is the second most common cancer in both sexes worldwide. Small-cell lung cancer (SCLC) is a form of neuroendocrine tumor, which is classified into limited and extensive-stage disease and shows excellent initial response to chemotherapy; however, almost all patients relapse later. During the past few years, several clinical trials have evaluated the effect of addition of immunotherapy to conventional chemotherapy in patients with extensive SCLC. Checkpoint inhibitors are currently under investigation, especially the CTLA-4 and PD-1/PD-L1 inhibitors. Nowadays, evidence show a statistically significant survival benefit of adding atezolizumab, an IgG1 monoclonal antibody targeting against PD-L1, to platinum-based chemotherapy plus etoposide in patients who have not received any previous systemic therapy. Furthermore, the role of nivolumab, an IgG4 anti-PD-1 monoclonal antibody, is significant for the treatment of relapsed SCLC cases. Recently, pembrolizumab was the first immunotherapeutic agent to be approved by the FDA for patients with metastatic SCLC with disease progression on or after platinum-based chemotherapy and at least one other prior line of chemotherapy. Nevertheless, prognostic biomarkers to immunotherapy response remain to be discovered.

## 1. Introduction

Lung cancer is a major cause of morbidity and mortality worldwide. Nowadays, it is the second most common cancer in both men and women [1]. Small-cell lung cancer (SCLC) represents about 10% to 15% of all lung cancers [1, 2]. It affects more frequently the Caucasian men and is strongly associated with tobacco consumption (98% of patients with SCLC have a smoking history) [3]. SCLC is categorized as neuroendocrine tumor (NET), and its subtypes include small-cell carcinoma and combined small-cell carcinoma (SCLC with a component of NSCLC) [4]. It is frequently associated with paraneoplastic syndromes, such as syndrome of inappropriate antidiuretic hormone secretion, Lambert–Eaton myasthenic syndrome, hypercalcemia, and many others [3, 4]. According to recent studies, it is characterised by multiple genetic alterations, reflecting genome instability. The majority of SCLCs express alterations in chromosome 3p and mutations regarding the following genes: RB1, TP53, RASSF1, MYC, FGFR1, and PTEN [5, 6]. Except for these genomic alterations, there is also malfunction of specific regulatory pathways.

SCLC is traditionally classified into limited-stage SCLC (LS-SCLC) and extensive-stage SCLC (ES SCLC) [7]. According to the latest IASLC staging system, extensive SCLC is defined as the disease which extends beyond one hemithorax at the time of initial diagnosis. Even though it is currently recommended to stage SCLC by the TNM classification, we refer to the ES/LS classification due to its usefulness in clinical decision making.

In general, SCLC is known for its aggressive behavior, rapid doubling time, growth, and early spread to distant sites; the median overall survival rates range from 15 to 20 months for limited stage disease and 8 to 13 months for extensive-stage disease [3, 8]. The overall survival depends mainly on the stage at the time of initial diagnosis. The 5-year survival rate is 20% to 25% for limited-stage disease but only about 2% for extensive-stage (ES) disease [9]. Currently, most patients are diagnosed after development of ES or even metastatic disease (around 70% of cases present with ES disease) [8, 9].

As far as therapy is indicated based on an acceptable performance status, SCLC shows high initial response to chemotherapy and radiation. First-line treatment for SCLC patients includes combination chemotherapy (cisplatin or carboplatin plus etoposide). Depending on the stage of the disease, radiation therapy may be added [8, 10]. Generally, first-line treatment results in a 60–80% overall response rate. However, all patients with ES disease and the majority of patients with limited-stage SCLC suffer relapse within months of completing initial therapy (platinum resistant is defined as relapse within 3 months and platinum sensitive ≥3–6 months), achieving a median progression-free survival (PFS) of only 5.5 months [5, 8]. Only a few patients will take clinically important advantage with second-line treatment. Clinical studies have shown that no therapy significantly improved the 15–20% response rate (RR) provided by second-line topotecan (the only drug with official approval in second line). Topotecan proved its efficacy regarding patients' response to therapy, safety, and symptom palliation in many phase II studies [11, 12]. The randomized multicenter phase III study on topotecan versus cyclophosphamide, doxorubicin, and vincristine (CAV) in patients with SCLC who progressed at least 60 days after completion of first-line therapy showed that topotecan was at least equally effective as CAV and led to its approval by the FDA [11, 12]. In addition, there are no consensus guidelines of care beyond second-line therapy.

Over the past decades, there has been a tremendous development in the survey of the pathogenesis of SCLC, leading to further understanding of the biological basis of the disease. Despite many clinical trials on new chemotherapy drugs and combinations and new biological agents, the overall survival rate has not significantly increased [9]. Nevertheless, in the past few years there are new advances in immunotherapy concerning SCLC. The aim of this article is to review recent developments and studies of immunotherapy that have been investigated for the treatment of extensive SCLC.

## 2. Materials and Methods

For this purpose, we searched current literature—using the PubMed search engine accessing the MEDLINE database, the Cochrane Library, and Medscape—for immunotherapy of extensive-stage SCLC. In particular, we used the following keywords: “Small cell lung cancer” AND “immunotherapy” AND “extensive stage” as an initial criterion. Articles published in the last 10 years were considered for review. Only articles in English were used. Mainly randomized studies were included in the present review. All titles and abstracts were screened for eligibility by two independent authors.

## 3. Results

### 3.1. Immunotherapy in SCLC: Immune Checkpoint Inhibitors

The high prevalence of paraneoplastic disorders among patients with SCLC led to the hypothesis that SCLC is an immunogenic disease. Generally, cancer is considered to overcome immune surveillance. Current evidence shows that SCLC is characterized by a characteristic neuroendocrine phenotype, expressing neural and endocrine markers [5]. Therefore new therapies that increase antitumor immune responses are being developed [4, 13].

Several immune checkpoint inhibitors are being evaluated in patients with SCLC. Immune checkpoint proteins are coinhibitory factors that diminish the antigen-specific immune response by reducing its potency [6]. In particular, the cytoxic T-lymphocyte-associated protein 4 (CTLA-4), the programmed death-1 (PD-1), and the CD47 pathways have been mostly studied.

It has been found that CTLA-4 promotes signals that suppress T-cell priming. As a result, treatment with antibodies specific for CTLA-4 could possibly restore an immune response through increased accumulation, survival of memory T cells, and depletion of Tregs [4, 8].

Similar to CTLA-4, PD-L1 is expressed on a number of cell types. Some examples are neoplastic and nonneoplastic cells within tumors. Interaction of PD-L1 with PD-1 on T cells leads to the deactivation of T cells and cytotoxicity and results in the exhaustion of T cells [4]. Recent studies have demonstrated that the expression of PD-L1 can be promoted by targeting the DNA damage response (DDR) proteins, the poly ADP-ribose polymerase (PARP), and also the checkpoint kinase 1 (CHK1) [4, 14].

Regarding the CD47 pathway, novel therapeutic strategies have shown promising results in clinical trials. In particular, CD47 is a cell surface molecule that promotes immune evasion [8]. It inhibits activation and phagocytic activity of macrophages by engaging signal-regulatory protein a (SIRPa) [4]. CD47 is also highly expressed on the surface of human SCLC cells.

### 3.2. The Use of Immunotherapy as First-Line Treatment in ES SCLC

Extensive SCLC is generally highly sensitive to chemotherapy. However, the development of resistance is eventually unavoidable. Immunotherapy, especially the introduction of immune checkpoint inhibitors and vaccine therapy, offers a new hope to patients with extensive SCLC [6].

Initially, a variety of cytokines have been investigated. According to recent studies, the addition of IFN-*α* provides no survival benefit in ES SCLC [15]. Afterwards, between December 2008 and January 2011, 213 patients were enrolled in a multicenter study that assessed the efficacy and safety of rilotumumab or ganitumab combined with etoposide and carboplatin or cisplatin as first-line treatment in patients with ES SCLC [16]. The study was a randomized, double-blind, placebo-controlled phase 1b/2. All eligible patients were over 18 years of age and had histologically or cytologically confirmed SCLC [16]. They also had extrathoracic metastases, malignant or pleural effusion, contralateral hilar adenopathy, or no limited disease. The study was divided to two parts. The primary endpoint for the phase 1b part was the incidence of dose-limiting toxicities, whereas the primary endpoint for the phase 2 was overall survival (OS), and the secondary endpoints included PFS, objective response rate, duration of response, safety, immunogenicity, and pharmacokinetic parameters [16]. Rilotumumab is a monoclonal antibody against hepatocyte growth factor (HGF). Ganitumab is a monoclonal antibody antagonist of insulin-like growth factor 1 receptor (IGF1R). Median OS was 10.8, 12.2 (hazard ratio (HR) = 0.84; 95% CI = 0.56–1.25; *p*=0.384), and 10.7 (HR = 0.95; 95% CI = 0.63–1.41; *p*=0.787) months for chemotherapy, chemo + rilotumumab, and chemo + ganitumab, respectively. Median PFS was 5.4 (HR = 1.05; 95% CI = 0.71–1.54; *p*=0.797) and 5.5 (HR = 1.05; 95% CI = 0.72–1.55; *p*=0.780) months, respectively [16]. The results showed that overall outcomes were not improved in patients with ES SCLC.

Furthermore, a study in the United Kingdom assessed the safety and efficacy of ipilimumab combined with standard first-line chemotherapy for extensive-stage SCLC [17]. The study was carried out from 2011 to 2014. It was a single arm, nonrandomized phase 2 study. The patients (42 in total) were men and women, had been diagnosed with ES SCLC, and had received no previous systemic therapy. The 1-year progression-free survival was chosen as the primary end point of the study [17]. Ipilimumab is an anti-CTLA4 antibody. At the same time, detection of autoantibodies was performed at baseline and during follow-up. Finally, the primary endpoint was not met. Nevertheless, there seemed to be an association with autoimmunity and benefit from ipilimumab with standard chemotherapy as first-line therapy in a subgroup of patients with ES SCLC [17].

A subsequent study investigated the potential benefit of the combined treatment of ipilimumab and standard chemotherapy (carboplatin/cisplatin plus etoposide) in patients with ES SCLC between 2012 and 2014 [18]. This study was a randomized, double-blind phase 2 trial. A number of 1,414 patients with documented ES SCLC without systemic therapy prior to the study were enrolled. All patients were adults, of both sexes. Overall survival among patients who received at least one dose of blinded study therapy was set as the primary endpoint [18]. Median OS was 11.0 months for chemotherapy plus ipilimumab versus 10.9 months for chemotherapy plus placebo (HR: 0.94; 95% CI, 0.81–1.09; *p*=0.3775). As a result, the addition of ipilimumab to etoposide and platinum did not improve OS compared with etoposide and platinum in chemotherapy-naive patients with ES SCLC.

### 3.3. Immunotherapy in Relapsed ES SCLC

In 2013 a multicenter phase 1/2 trial was conducted (CheckMate 032). 216 patients of both sexes over the age of 18 were enrolled in the study during the period of 2013 to 2015 [19]. The patients had limited- or extensive-stage SCLC and were diagnosed by disease progression after at least one previous platinum-containing line of therapy. Patients were treated with nivolumab or nivolumab plus ipilimumab [19]. Nivolumab is a human IgG4 PD-1 immune checkpoint inhibitor antibody. According to preclinical evidence, the combined therapy with PD-1 and CTLA-4 receptor blockade could probably improve antitumor activity by complementing each other by the release of tumor antigens because of chemotherapy, followed by their presentation to T cells by antigen-presenting cells [6]. The proportion of patients with a confirmed objective response was determined as the primary endpoint of the trial [19]. This was defined as the number of patients with a best overall response as per investigator assessed RECIST criteria divided by the number of assigned patients. The secondary endpoints included overall survival, progression-free survival, duration of response, and the occurrence of treatment-related adverse events leading to treatment discontinuation [19]. The outcomes of the study showed that nivolumab monotherapy and nivolumab plus ipilimumab provide clinically meaningful activity and an acceptable safety profile for patients with extensive-stage SCLC and disease progression after at least one previous platinum-containing regimen [19]. Main study limitations were the nonrandomization and that it was not powered for formal comparisons across cohorts. The study “CheckMate 032” proved an overall response rate (ORR) of 10% with Nivolumab and 23% with nivolumab (1 mg/kg) plus ipilimumab (3 mg/kg), with grade 3-4 adverse effects of 14% and 33% in nivolumab and nivolumab plus ipilimumab, respectively [19, 20].

### 3.4. IMpower133: The First Trial with Positive Results in First-Line Treatment of ES SCLC

Moreover, the trial “IMpower133” evaluated the efficacy and safety of the addition of atezolizumab to conventional chemotherapy (carboplatin plus etoposide) in patients with extensive-stage small-cell lung cancer who had not previously received treatment [21]. Atezolizumab is a humanized monoclonal antibody of IgG1 isotype against the PD-L1 protein. This was a phase 3, double-blinded, randomized, placebo-controlled trial. Eligible patients were patients of both sexes, aged over 18 years, who were histologically or cytologically diagnosed by ES SCLC. No patient had previously received systemic treatment [21]. Also, patients with treated asymptomatic brain metastases were included. Between 2016 and 2017, 403 patients were enrolled in the trial. The primary endpoints were OS and investigator-assessed PFS. As for the key secondary endpoints, the objective response rate, the duration of response, and safety of the therapy were taken into consideration [21]. Results showed that the addition of atezolizumab to carboplatin and etoposide provided a significant improvement in OS and PFS, compared with carboplatin and etoposide alone in 1L ES SCLC (mOS: 12.3 vs. 10.3 months; HR: 0.70 (*p*=0.0069); 12-month OS: 51.7% vs. 38.2%; mPFS: 5.2 vs. 4.3 months; HR: 0.77 (*p*=0.017); 12-month PFS: 12.6% vs. 5.4%). Furthermore, the safety profile of atezolizumab plus chemotherapy was comparable to chemotherapy alone [21]. As a conclusion, atezolizumab plus carboplatin + etoposide could be the new standard of care for the first-line treatment of ES SCLC.

Despite the promising results of the study, there are a number of limitations that should be taken into account before the use of atezolizumab plus chemotherapy in everyday clinical practice [21]. Firstly, the role of atezolizumab has not been explored among patients with asymptomatic untreated brain metastases [21]. Secondly, the results regarding patients with treated brain metastases need further investigation due to their small participation in the trial. Thirdly, patients with active autoimmune diseases were excluded from the study. Furthermore, future analysis is needed to investigate the imbalance in overall survival among younger patients and the possible predictive role of blood-based TMB levels [21]. Also, the precise mechanism of the action of atezolizumab has not yet been clarified.

### 3.5. Ongoing Trials in ES SCLC

Based on the encouraging data derived from the evaluation of immune checkpoint inhibitors, several trials are currently ongoing. Firstly, the “CheckMate 331” trial is a phase 3 randomized study [22]. The purpose of this study is to assess the use of nivolumab along with chemo-topotecan in case of relapsed SCLC. It is a randomized trial and has determined OS as its primary endpoint [22]. Another ongoing trial is the “CheckMate 451” trial, which is attempting to assess the OS and PFS of 940 patients treated with nivolumab as monotherapy or with the addition of ipilimumab as first-line maintenance therapy [23].

In addition to the previous ongoing studies, the “Keynote 028” is a 1b phase trial aiming to evaluate the potential role of pembrolizumab in the therapy of patients with ES SCLC. Eligible patients have previously received systemic treatment and present with >1% PD-L1 expression [24]. Pembrolizumab is an IgG4 isotype antibody that targets the PD-1 receptor of lymphocytes and enhances immune response to cancer evasion. The primary endpoint of the study is ORR. Safety, PFS, and OS are some of the secondary outcomes. Early results also indicate promising antitumor activity in patients with PD-L1-positive SCLC who have progressed on prior platinum-based therapy [24]. The “Keynote 158” is also a phase 2 study, targeting to prove the beneficial effect of pembrolizumab among patients with ≥1% PD-L1 expression. Tumors were PD-L1 positive in 42 patients (39%) and PD-L1 negative in 50 patients (47%). ORR of 18% was observed overall with 35% in PD-L1 positive tumors and 6% in PD-L1 negative tumors [25]. Median PFS was 2 months in all patients. The secondary outcome was safety of pembrolizumab.

On June 17, 2019, the FDA approved the use of pembrolizumab for patients with metastatic SCLC with disease progression on or after platinum-based chemotherapy and at least one other prior line of therapy [26]. This decision was based on the early results of the trials “Keynote 028” and “Keynote 158”. It is the first time that an immunotherapeutic agent has been approved as monotherapy for the treatment of patients with SCLC. The recommended dose is 200 mg administered intravenously [26]. In particular, the FDA approval followed the observed tumor RR and the duration of response regarding 83 patients who participated in 1 of 2 of the previous trials [24, 25]. These patients were reported with disease progression on or after two or more previous lines of therapy. During the trial they received either pembrolizumab 200 mg intravenously every 3 weeks (*N* = 64) or 10 mg/kg intravenously every 2 weeks (*N* = 19). Treatment was discontinued in case of confirmed disease progression, unacceptable toxicity, or a maximum of 24 months. According to the results, the ORR was 19%, and the complete RR was 2%. The duration of response was ≥6 months in 94% of the patients and ≥18 months in 56% of them. As for the toxicity of the agent, serious adverse reactions were reported in 31% of the patients and included pneumonia and pleural effusion [26].

Another ongoing study of pebrolizumab for SCLC is the “Keynote 604” trial which is quite similar with IMpower133. It focuses on the OS and PFS of patients with ES SCLC who have not received any kind of therapy [27]. Eligible patients have no treated CNS metastases. A total of 430 patients were enrolled in the study and randomized to therapy with pembrolizumab plus carboplatin/cisplatin and etoposide versus therapy with placebo plus carboplatin/cisplatin and etoposide. The results are expected. Other ongoing studies are the “Caspian” and the “Meru” trials. Both of them are currently undergoing phase 3 trials. The “Caspian” investigates the effect of therapy with durvalumab among naïve patients with ES SCLC [28]. Durvalumab is an FDA-approved IgG1κ monoclonal antibody against PD-1/PD-L1 ligand. Eligible patients are confirmed with ES SCLC diagnosis, suitable to receive platinum-based chemotherapy as first-line treatment [28]. Exclusion criteria are active, symptomatic CNS metastases, and previous immune-mediated therapy. In total, 795 patients will be randomized to therapy with durvalumab plus tremelimumab plus conventional chemotherapy versus durvalumab plus standard chemotherapy versus standard chemotherapy. The primary endpoints include PFS and OS.

Regarding the “Meru” trial, it is a phase 3 study investigating the use of Rova-T in 740 patients with CR, PR, or SD after of 4 cycles of first-line platinum-doublet chemotherapy. The primary endpoints are PFS and OS. Rovalpituzumab tesirine (Rova-T) is an experimental antibody-drug conjugate targeting the protein DLL3 on tumor cells. In the past, the results of the phase II TRINITY trial on the use of Rova-T in the third-line setting for patients with relapsed SCLC showing high DLL3 expression were disappointing (ORR = 16%, median OS = 5.6 months) [29].  Table 1 summarizes most important clinical trials regarding immunotherapy agents for SCLC.

## 4. Discussion

### 4.1. Critical Approach

Immunotherapy is a revolutionizing cancer treatment among patients with ES SCLC. Initial studies of ipilimumab, a CTLA-4 antibody, suggested a benefit, but it was not confirmed in different phase randomized trials [30]. Even though the expression of PD-L1 is generally low in SCLC, the checkpoint inhibitor nivolumab was approved by the FDA as first immunotherapy agent for SCLC due to the “Checkmate 032” clinical trial. Nowadays, it is used in patients with relapsed ES SCLC [30]. Following the “Checkmate 032” trial, the “Impower133” trial proved that the addition of atezolizumab to chemotherapy as first-line treatment can improve OS. This ground-breaking outcome should remain under investigation. A possible explanation of the effectiveness of atezolizumab when it is combined with conventional chemotherapy is the fact that carboplatin and etoposide cannot deplete the intratumoral T cell population, which happen to be the targets of atezolizumab. At any case, the results of several ongoing and future trials have to be counted in.

### 4.2. Biomarkers

The fact that only a number of patients with ES SCLC show memorable response to specific targeted immunotherapy strategies has created the need for discovery of novel biomarkers in order to predict the population of patients most likely to benefit from them [31]. The predictive role of TMB to ICBs has been investigated in the “Checkmate 032” trial. The tumor mutational burden (TMB) is high in SCLC. Patients with a high overall TMB in combination with a heavy tobacco exposure history appear to benefit from immunotherapy the most [9, 20]. There was a difference in RR for both nivolumab monotherapy (a 4.8% RR for those with low TMB compared with 21.3% for those with a high TMB) or combination therapy (22.2% compared with 46.2%) during the previous trial. In addition, 1 yr PFS was prolonged for both therapies, and 1 yr OS was significantly prolonged for the combination in those with a high TMB [9]. In contrast to TMB, PD-L1 was positive in 12% of the patients and was not predictive of response.

### 4.3. Future Therapeutic Approaches

Although various targets except for PD- L1/PD1, CTLA- 4, and CD47 have been identified, they remain in early stages of clinical investigation and development [20]. Some examples are the following: BTLA, VISTA, LAG3, and TIM-3. Recent studies show also that the combination of immunotherapy with radiation could possibly enhance the overall therapeutic benefit in patients with ES SCLC [32]. In particular, radiation is assumed to be immunomodulatory, as it can increase tumor antigen production and presentation and upregulate cytotoxic T-lymphocyte activity [33]. Innovative trials on the potential synergy of radiotherapy with immune therapy in patients with ES SCLC are expected in the near future.

## 5. Conclusions

Extensive small-cell lung cancer is a rapidly growing tumor with distant metastases and extremely poor prognosis. Besides conventional chemotherapy, which provides encouraging results at first, novel therapeutic strategies are under investigation. The immunotherapy plays a significant role in the treatment of ES SCLC. Recently, pembrolizumab was the first immunotherapeutic agent to be approved by the FDA for patients with metastatic SCLC with disease progression on or after platinum-based chemotherapy and at least one other prior line of therapy. Immunotherapy remains a promising approach despite the outcomes of the first trials, which failed to meet their endpoints. A review of current evidence highlights the urge for the specific biomarkers to be found in order to achieve better and personalized results.

## Figures and Tables

**Table 1:** Selected clinical trial of immunotherapy agents for SCLC.

Trial	Phase	Line	Study type	Therapy	Primary endpoint	Results	ClinTrial Gov Identifier
Checkmate 032 [19]	I/II	3^rd^	Randomized cohort	Nivolumab ± ipilimumab	ORR	Nivo 3 mg/kg ORR: 12%	NCT01928394
						Nivo 1 mg/kg + ipi 3 mg/kg ORR: 23%	
						Nivo 3 mg/kg + ipi 1 mg/kg ORR: 19%	

IMpower 133 [21]	III	1^st^	Randomized, multicenter double-blind, placebo-controlled	Carboplatin + etoposide + atezolizumab or carboplatin + etoposide + placebo	PFS + OS	Median OS: 12.3 months vs 10.3 months	NCT02763579
						Median PFS: 5.2 months vs 4.3 months	

Checkmate 331 [22]	III	2^nd^	Randomized, open label, global	Nivolumab vs topotecan/amrubicin	OS	Primary endpoint not met	NCT02481830

Checkmate 451 [23]	III	1^st^ (main)	Randomized, double-blind, multicenter	Nivolumab, nivolumab + ipilimumab, placebo	OS	Primary endpoint not met	NCT02538666

Keynote 028 [24]	Ib	2^nd^	Open label, nonrandomized, multicenter, multicohort	Pembrolizumab	Best OR (RECIST version 1.1)	ORR: 33%	NCT02054806

Keynote 158 [25]	II	2^nd^	Open label, nonrandomized, multicenter, multicohort	Pembrolizumab	ORR	ORR: 19%	NCT02628067

Keynote 604 [27]	III	1^st^	Randomized, double-blind, placebo-controlled	Pembrolizumab + platinum/etoposide vs platinum/etoposide	PFS + OS	Ongoing	NCT03066778

Caspian [28]	III	1^st^	Randomized, multicenter, open label	Durvalumab ± tremelimumab + chemotherapy vs chemotherapy	PFS + OS	Ongoing	NCT03043872

Meru [29]	III	2^nd^	Randomized, double-blind, placebo-controlled	Rova-T + dexamethasone vs placebo (after chemo)	PFS + OS	Ongoing	NCT03033511
